# Differentiation of pulmonary tuberculosis from non-tuberculous solid lung lesions using radiomics and clinical-semantic features on contrast-enhanced CT

**DOI:** 10.3389/fmed.2026.1754750

**Published:** 2026-03-31

**Authors:** Sunyi Zheng, Jing Wang, Jing Liang, Xiaomeng Yang, Jiaxin Liu, Yanju Li, Pengcheng Wei, Jianyu Xiao, Jaeyoun Yi, Jianwei Wang, Zhaoxiang Ye, Xiaonan Cui

**Affiliations:** 1 National Clinical Research Center for Cancer, Tianjin’s Clinical Research Center for Cancer, State Key Laboratory of Druggability Evaluation and Systematic Translational Medicine, Tianjin Key Laboratory of Digestive Cancer, Key Laboratory of Cancer Prevention and Therapy, Medical Artificial General Intelligence for Computation (MAGIC) Lab, Department of Radiology, Tianjin Medical University Cancer Institute and Hospital, Tianjin, China; 2 School of Public Health, Tianjin University of Traditional Chinese Medicine, Tianjin, China; 3 Department of Radiology, Baoding No. 6 Hospital, Baoding, Hebei, China; 4 Coreline Soft, Seoul, Republic of Korea; 5 Department of Diagnostic Radiology, National Cancer Center, National, Clinical Research Center for Cancer/Cancer Hospital, Chinese Academy of Medical Sciences and Peking Union Medical College, Beijing, China

**Keywords:** computed tomography, machine learning, pulmonary tuberculosis, radiomics, solid lung lesion

## Abstract

**Background:**

To explore the feasibility of a combined model integrating radiomics and clinical-semantic features for differentiating pulmonary tuberculosis (PTB) from non-tuberculous solid lung lesions on contrast-enhanced CT.

**Methods:**

In this study, 900 patients enrolled before October 2016 were randomly partitioned into training and internal validation sets at a 3:1 ratio, while those recruited between October 2016 and October 2017 formed an independent temporal validation set. Clinical-semantic features were selected through univariate analysis followed by multivariate analysis, while predictive radiomics features were identified using analysis of variance, Spearman correlation analysis, least absolute shrinkage and selection operator regression. Binary logistic regression was then used to construct the clinical-semantic, radiomics, and combined models. Model performance was evaluated using average precision (AP) derived from the precision-recall curve, and differences between models were assessed using bootstrap resampling. Clinical utility was assessed using decision curve analysis.

**Results:**

Following feature selection, two clinical-semantic and three radiomics features were incorporated into the combined model. This model achieved APs of 0.91, 0.85, and 0.62 in the training, internal validation, and temporal validation sets, respectively, outperforming the clinical-semantic model, which yielded APs of 0.64, 0.61, and 0.41 (*p* < 0.001, *p* < 0.001, and *p* = 0.006). The radiomics model also outperformed the clinical-semantic model across the three sets with APs of 0.88, 0.82, and 0.45. Decision curve analysis showed that the combined model can provide good net benefit across varying threshold probabilities.

**Conclusion:**

By integrating clinical-semantic and radiomics features, the combined model enables accurate differentiation between PTB and non-tuberculous solid lung lesions, potentially facilitating non-invasive diagnosis and personalized treatment planning.

## 1 Introduction

Tuberculosis remains a global health burden, ranking among the top infectious causes of morbidity and mortality worldwide (1). As a highly contagious airborne disease, tuberculosis poses a serious threat of community spread, highlighting the importance of early diagnosis and timely intervention to disrupt the transmission chain. Pulmonary tuberculosis (PTB) is the most common form and frequently present as solid nodules or masses on imaging (2). However, such radiologic appearances are not specific to PTB. In clinical settings, solid pulmonary lesions are also observed in a range of non-tuberculous conditions, including primary lung cancers, as well as benign or inflammatory diseases such as inflammatory pseudotumors. The overlapping imaging features between PTB and these non-tuberculous entities create a diagnostic dilemma. Such a difficulty in differentiation can lead to important clinical consequences, as the underlying causes require fundamentally different management strategies. For instance, PTB is typically treated with prolonged courses of anti-tuberculous medications and supported by public health measures to limit transmission (3). In contrast, solid lesions caused by malignancies or inflammatory conditions often require surgical intervention, immunomodulatory treatment, or cancer-specific therapies. Failure to distinguish PTB from other conditions may result in delayed or inappropriate treatment, unnecessary invasive procedures, and increased risk of disease transmission.

Current approaches for diagnosing PTB rely primarily on microbiological and histopathological evaluation. Sputum smear microscopy and *mycobacterium tuberculosis* culture remain the mainstay for bacteriological confirmation (4). Although culture is considered the gold standard, it is time-consuming and often yields false negatives in cases with low bacillary load. Invasive procedures such as CT-guided percutaneous needle biopsy can provide histopathological evidence and help rule out malignancy or other differential diagnoses, yet these methods are associated with procedural risks such as bleeding and pneumothorax (5). As a non-invasive modality, contrast-enhanced CT is widely used to evaluate pulmonary lesions, providing information on lesion morphology and additional enhancement patterns compared to non-contrast CT (6–8). However, PTB lesions that present as solid nodules or masses frequently overlap in appearance with malignant tumors or benign inflammatory conditions. The diagnostic ambiguity underscores the need for more objective and accurate imaging-based tools to support clinical decision-making.

Radiomics has emerged as a promising approach for extracting quantitative features from standard medical images, transforming visual information into objective imaging biomarkers (9, 10). By enabling detailed characterization of pulmonary lesions, radiomics has shown encouraging results in differentiating malignant from benign nodules, identifying tumor subtypes, predicting treatment response and prognosis (11–18). In addition to oncologic applications, researchers have also explored the use of radiomics on contrast-enhanced CT scans in the identification of PTB. For example, the study of Yang et al. showed radiomics was able to distinguish PTB from lung adenocarcinoma, while Zhao et al. found radiomics to be useful in distinguishing PTB from non-neoplastic inflammatory conditions such as organizing pneumonia and fungal infections (19, 20). However, previous studies mainly focused on specific comparison groups and were therefore limited in their clinical scope. In routine clinical practice, pulmonary tuberculosis may present as a solid lung lesion that closely resembles a wide spectrum of non-tuberculous conditions, including both benign and malignant tumors, making accurate differential diagnosis challenging. To date, the feasibility of radiomics for differentiating PTB from a broad range of non-tuberculous solid lung lesions, including both benign and malignant tumors, has not been investigated.

To address this gap, this study aims to develop and validate a radiomics model based on contrast-enhanced CT for distinguishing PTB from non-tuberculous solid lung lesions, and to determine whether combining radiomic and clinical-semantic features enhances diagnostic performance.

## 2 Materials and methods

### 2.1 Study patients

This retrospective study was performed in accordance with the Declaration of Helsinki and approved by the Institutional Review Board and Ethical Committee of Tianjin Medical University Cancer Institute and Hospital. Data from 924 patients were retrospectively collected between April 2012 and October 2017. The dataset is private due to patient confidentiality and institutional data-sharing policies. The inclusion criteria were as follows: (a) patients had a primary solitary solid lung lesion with a diameter ≥8 mm on CT images; (b) patients underwent preoperative contrast-enhanced chest CT examinations; and (c) patients had available postoperative pathological results. The exclusion criteria were: (a) poor-quality CT images; (b) absence of CT images; (c) receipt of preoperative therapy; (d) incomplete clinical data or ambiguous pathological diagnosis; and (e) a history of other malignant tumors.

### 2.2 Pathological evaluation

Two pathologists independently assessed the pathological specimens using the fifth edition of the WHO Classification of Thoracic Tumors (21), with any discrepancies resolved through discussion. Based on this classification, the histologic types of lung lesions included tuberculosis, adenocarcinoma, squamous cell carcinoma, large cell carcinoma, pulmonary hamartoma, sclerosing hemangioma, and inflammatory pseudotumor. These types were stratified into pulmonary tuberculosis and non-tuberculosis solid lung lesions.

### 2.3 Radiomics features from segmented tumors

Tumor segmentation was performed semi-automatically using the 3D Slicer software (version 5.6.2) by two experienced radiologists. Following the initial segmentation, the results were reviewed and manually adjusted on contrast-enhanced CT images by a senior radiologist to ensure accuracy. Before radiomics feature extraction, all CT data was resampled to 1 × 1 × 1 mm voxel size to ensure consistent spatial resolution. Radiomics features were then extracted using PyRadiomics version 3.0.1 from both the original images and a series of transformed images processed with wavelet, Laplacian of Gaussian, gradient, logarithmic, square, square root, exponential, two-dimensional and three-dimensional local binary pattern filters. The extracted 1834 features encompassed first-order statistics, shape descriptors, and various texture matrices, including the gray level co-occurrence matrix, gray level run length matrix, gray level size zone matrix, gray level dependence matrix, and neighboring gray tone difference matrix. A fixed bin width of 25 HU was applied for grey-level discretization to ensure consistency in texture feature extraction across scans. To evaluate feature robustness, a random sample of 50 cases was selected for reproducibility testing using the intraclass correlation coefficient. One month later, the same subset was independently re-segmented by both radiologists to assess intra- and inter-observer consistency. Features with an intraclass correlation coefficient (ICC) above 0.75 were deemed reliable and retained for further analysis.

### 2.4 CT examination and clinical-semantic features

Chest CT scans were performed using Discovery CT750HD, LightSpeed 16, and Somatom Sensation 64 CT devices. The scanning range was from the lung apex to below the diaphragm. The tube voltage was 120 kV, and the tube current was automatically adjusted. The reconstruction thickness was 1.25 mm with a spacing of 0.984 mm in GE CT systems, whereas the reconstruction thickness was 1.5 mm with a spacing of 0.95 mm for Siemens CT devices.

CT characteristics of lung lesions were independently evaluated and measured by two senior thoracic radiologists with 11 and 16 years of clinical experience in chest imaging, respectively. The radiologists were blinded to both the pathological diagnosis and the study objective. In cases of disagreement, final decisions were made through discussion and consensus by the radiologists. A total of two clinical features and fourteen semantic imaging features were included in the analysis. These features included age, sex, lesion location, long-axis diameter, short-axis diameter, shape, calcification, necrosis, cavitation, air bronchograms, pleural indentation, vascular invasion, postobstructive pneumonia, satellite nodules, pleural effusion and lymph node enlargement.

### 2.5 Model development

In this study, we developed three models which include radiomics, clinical-semantic, and combined models for the differentiation of PTB from non-tuberculous lung solid lesions.

Prior to model construction, all radiomic features were standardized using Z-score normalization to reduce bias arising from differences in feature scales. The mean and standard deviation for each feature were calculated exclusively from the training set and subsequently applied to normalize the training and validation sets, thereby preventing information leakage during model development. The feature selection process was conducted in four consecutive stages. Initially, features with an intraclass correlation coefficient below 0.75 were discarded to ensure measurement consistency. Next, one-way analysis of variance (ANOVA) was employed to eliminate features lacking statistical relevance to the clinical outcome, applying a threshold of 0.1. In the third step, Spearman correlation analysis was performed to identify redundant features. For any pair of features with a correlation coefficient greater than 0.7, the one with less clinical or statistical relevance was removed to avoid multicollinearity. Finally, the least absolute shrinkage and selection operator (LASSO) regression with ten-fold cross-validation was implemented to identify the most informative features for model construction. The final binary logistic regression model with backward selection was trained with class-weight balancing to mitigate the effect of sample imbalance. In the class-weight balancing strategy, the weight assigned to each class is inversely proportional to its frequency in the training data. This increases the penalty for misclassifying the minority class and helps mitigate the bias toward the majority class during model training. To ensure model integrity and prevent data leakage, all feature selection steps including ANOVA, Spearman correlation, and LASSO were performed strictly and exclusively on the training set.

To develop the clinical-semantic model, logistic regression was also employed. Initially, univariate analysis was performed to identify potential predictors, with variables showing a *p*-value less than 0.1 considered for further evaluation. These candidates were then entered into a multivariate logistic regression model with backward likelihood ratio selection and class weighting to identify independent predictors and finalize the model.

After developing separate models based on radiomics and clinical-semantic features, we constructed a combined model for PTB prediction. Specifically, the optimal features from the final radiomics and clinical-semantic models were combined into a single feature set and used for logistic regression modeling with backward elimination. To mitigate the impact of sample imbalance, the class-weight balancing strategy was also applied during model development. Based on the final logistic regression model, a nomogram was constructed to provide an individualized estimate of the probability of PTB.

### 2.6 Statistical analysis

Statistical analyses were conducted using Python (version 3.10.11) and R (version 4.4.2). Radiomics features were extracted using PyRadiomics (version 3.0.1). Categorical variables were summarized as counts and percentages, whereas continuous variables were expressed as means with corresponding standard deviations. Group comparisons between PTB and non-tuberculosis cohorts were performed using the chi-square test for categorical variables (22). For continuous variables, either the independent-samples t-test or the Wilcoxon rank-sum test was employed, based on the distribution characteristics of the data (23). Model performance was evaluated using average precision (AP), the area under the receiver operating characteristic curve (AUC), sensitivity, specificity, positive predictive value (PPV), negative predictive value (NPV), F1 score and balanced accuracy. The optimal cutoff probability was determined by the maximum Youden index on the training set’s receiver operating characteristic curve. Once determined, the optimal cutoff was fixed and applied unchanged to the internal and temporal validation sets to calculate performance metrics, ensuring that the evaluation remained independent and free from information leakage. Differences in AP between models were evaluated using bootstrap resampling. AUC values were reported with 95% confidence intervals on training and internal validation sets. Statistical differences in AUC between models were examined using the DeLong test (24). Decision curve analysis was performed to assess the clinical utility of the models by quantifying the net benefit across a range of threshold probabilities. The Hosmer-Lemeshow test was used to evaluate the goodness-of-fit of the logistic regression models by comparing the predicted probabilities with the observed outcomes across deciles of risk (25). A two-tailed *p*-value < 0.05 was considered indicative of statistical significance. To provide a more rigorous validation, we also incorporated the Brier score, calibration slope, and calibration intercept for all sets. For model interpretability, the Shapley Additive Explanations (SHAP) method was used to determine the relative importance of individual radiomic features, offering a visual understanding of how each feature contributed to the prediction outcomes (26). The CLEAR and METRICS checklists are provided as Supplementary material, and the manuscript has been written with reference to the RQS 2.0 tool to enhance transparency and reporting quality (27–29).

## 3 Results

### 3.1 Patient information

**Figure 1:**
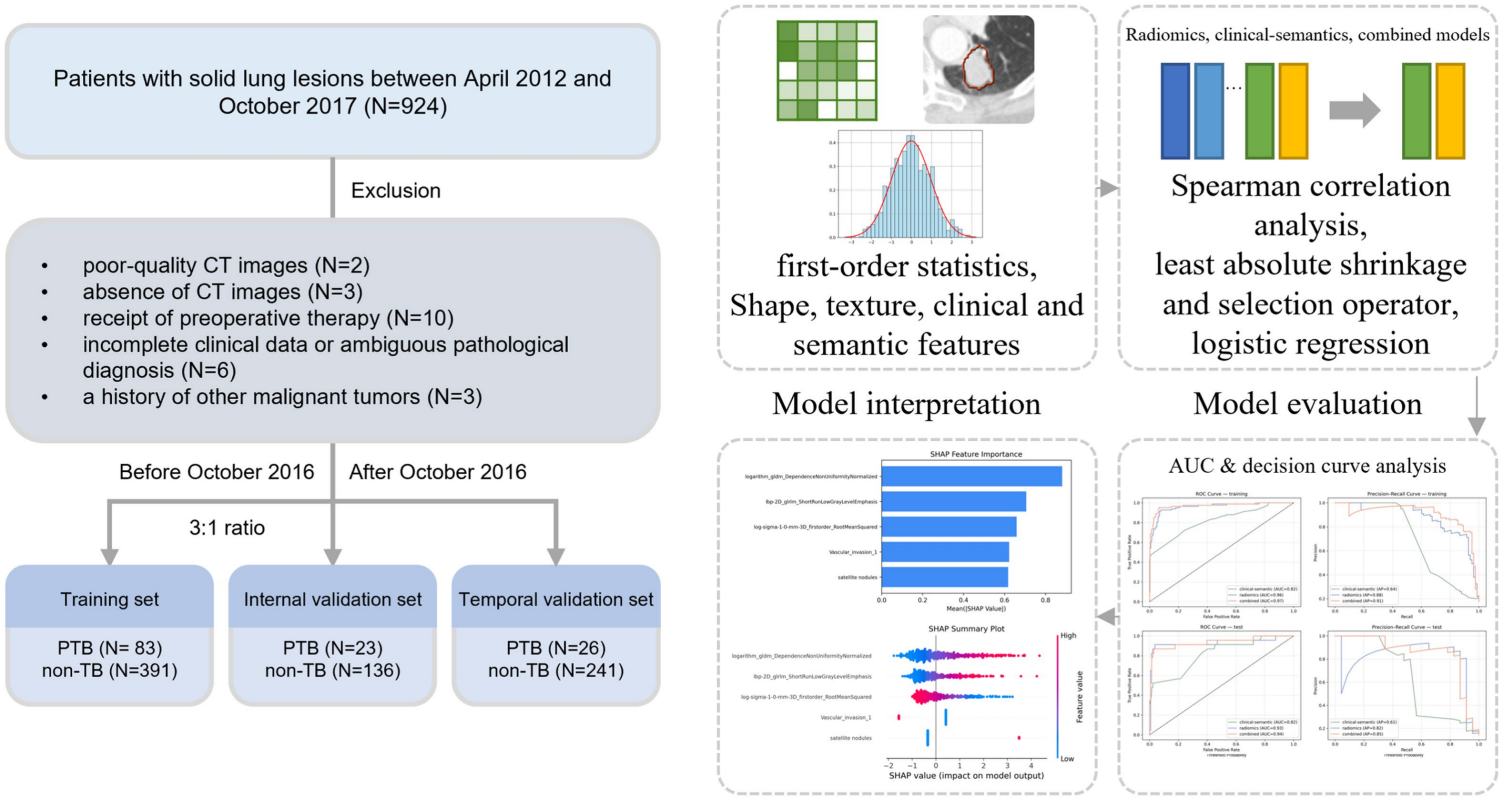
Patient selection process and study workflow. PTB represents the pulmonary tuberculosis group, and non-TB represents the group with non-tuberculous solid lung lesions. After feature extraction and selection, models were constructed using logistic regression. The areas under the receiver operating characteristic and precision-recall curves were used for model evaluation, and SHapley additive explanations was applied to enhance model interpretability.

After excluding patients with a history of cancer, preoperative therapy, poor image quality, absence of CT images, or incomplete clinicopathological information, a total of 900 patients were enrolled in the study. The patient selection process is illustrated in Figure 1. Among the 900 patients, those enrolled before October 2016 were randomly divided into training and internal validation sets at a 3:1 ratio, while those recruited between October 2016 and October 2017 served as the temporal validation set. Across the three sets, PTB patients were generally younger than non-tuberculosis patients, with mean ages of 52.88 ± 9.74, 55.91 ± 10.90, and 53.42 ± 8.72, compared to 57.75 ± 9.35, 56.27 ± 8.18, and 59.24 ± 8.29 in the non-PTB groups. The majority of PTB patients were female, accounting for 69.9, 56.5, and 61.5% of the training, internal validation, and temporal validation sets, respectively. Peripheral lesions were the predominant location in both PTB and non-tuberculosis groups. Compared with non-tuberculosis lesions, PTB lesions were smaller in size, with both long- and short-axis diameters showing consistent differences across the two groups (all *p* < 0.05). Furthermore, PTB lesions were more frequently associated with the absence of necrosis, lack of vascular invasion and lack of lymph node enlargement. Detailed patient characteristics of PTB and non-tuberculosis cases for sets are summarized in Table 1.

**Table 1:** Clinical-semantic features of patients in the training, internal validation and temporal validation sets.

Clinical-semantic features	Training set (*n* = 474)			Internal validation set (*n* = 159)			Temporal validation set (*n* = 267)		
	Non-TB (*n* = 391)	PTB (*n* = 83)	*p* value	Non-TB (*n* = 136)	PTB (*n* = 23)	*p* value	Non-TB (*n* = 241)	PTB (*n* = 26)	*p*-value
Age (years)	57.75 ± 9.35	52.88 ± 9.74	<0.001	56.27 ± 8.18	55.91 ± 10.90	0.854	59.24 ± 8.29	53.42 ± 8.72	0.001
Sex			0.028			1.000			0.668
Male	169 (43.2%)	25 (30.1%)		60 (44.1%)	10 (43.5%)		82 (34.0%)	10 (38.5%)	
Female	222 (56.8%)	58 (69.9%)		76 (55.9%)	13 (56.5%)		159 (66.0%)	16 (61.5%)	
Lesion location			0.028			0.046			0.007
Peripheral	346 (88.5%)	80 (96.4%)		114 (83.8%)	23 (100.0%)		176 (73.0%)	25 (96.2%)	
Central	45 (11.5%)	3 (3.6%)		22 (16.2%)	0 (0.0%)		65 (27.0%)	1 (3.8%)	
Long-axis diameter (mm)	30.95 ± 14.92	23.94 ± 9.97	<0.001	32.18 ± 16.01	22.13 ± 10.07	0.004	30.49 ± 15.56	21.04 ± 7.89	0.003
Short-axis diameter (mm)	24.34 ± 11.79	17.95 ± 6.68	<0.001	25.39 ± 12.16	16.74 ± 7.05	0.001	23.84 ± 12.71	15.85 ± 6.28	0.002
Shape			0.226			0.116			1.000
Round	210 (53.7%)	38 (45.8%)		73 (53.7%)	8 (34.8%)		118 (49.0%)	13 (50.0%)	
Irregular	181 (46.3%)	45 (54.2%)		63 (46.3%)	15 (65.2%)		123 (51.0%)	13 (50.0%)	
Calcification			0.033			0.549			0.478
No	335 (85.7%)	63 (75.9%)		114 (83.8%)	18 (78.3%)		221 (91.7%)	23 (88.5%)	
Yes	56 (14.3%)	20 (24.1%)		22 (16.2%)	5 (21.7%)		20 (8.3%)	3 (11.5%)	
Necrosis			0.023			0.169			0.011
No	329 (84.1%)	78 (94.0%)		106 (77.9%)	21 (91.3%)		195 (80.9%)	26 (100.0%)	
Yes	62 (15.9%)	5 (6.0%)		30 (22.1%)	2 (8.7%)		46 (19.1%)	0 (0.0%)	
Cavitation			0.014			0.511			1.000
No	334 (85.4%)	61 (73.5%)		119 (87.5%)	19 (82.6%)		198 (82.2%)	22 (84.6%)	
Yes	57 (14.6%)	22 (26.5%)		17 (12.5%)	4 (17.4%)		43 (17.8%)	4 (15.4%)	
Air bronchograms			0.873			0.770			<0.001
No	324 (82.9%)	68 (81.9%)		110 (80.9%)	20 (87.0%)		161 (66.8%)	26 (100.0%)	
Yes	67 (17.1%)	15 (18.1%)		26 (19.1%)	3 (13.0%)		80 (33.2%)	0 (0.0%)	
Pleural indentation			0.894			0.338			0.008
No	113 (28.9%)	23 (27.7%)		45 (33.1%)	5 (21.7%)		91 (37.8%)	3 (11.5%)	
Yes	278 (71.1%)	60 (72.3%)		91 (66.9%)	18 (78.3%)		150 (62.2%)	23 (88.5%)	
Vascular invasion			0.004			0.533			0.056
No	311 (79.5%)	77 (92.8%)		115 (84.6%)	21 (91.3%)		207 (85.9%)	26 (100.0%)	
Yes	80 (20.5%)	6 (7.2%)		21 (15.4%)	2 (8.7%)		34 (14.1%)	0 (0.0%)	
Postobstructive pneumonia			<0.001			0.003			0.078
No	288 (73.7%)	76 (91.6%)		88 (64.7%)	22 (95.7%)		165 (68.5%)	13 (50.0%)	
Yes	103 (26.3%)	7 (8.4%)		48 (35.3%)	1 (4.3%)		76 (31.5%)	13 (50.0%)	
Satellite nodules			<0.001			<0.001			<0.001
No	389 (99.5%)	44 (53.0%)		133 (97.8%)	11 (47.8%)		236 (97.9%)	15 (57.7%)	
Yes	2 (0.5%)	39 (47.0%)		3 (2.2%)	12 (52.2%)		5 (2.1%)	11 (42.3%)	
Pleural effusion			0.634			0.604			1.000
No	385 (98.5%)	81 (97.6%)		128 (94.1%)	23 (100.0%)		236 (97.9%)	26 (100.0%)	
Yes	6 (1.5%)	2 (2.4%)		8 (5.9%)	0 (0.0%)		5 (2.1%)	0 (0.0%)	
Lymph node enlargement			0.008			0.169			0.275
No	309 (79.0%)	76 (91.6%)		106 (77.9%)	21 (91.3%)		196 (81.3%)	24 (92.3%)	
Yes	82 (21.0%)	7 (8.4%)		30 (22.1%)	2 (8.7%)		45 (18.7%)	2 (7.7%)	

The PTB group refers to patients with pulmonary tuberculosis, while the non-TB group refers to those with non-tuberculous solid lung lesions.

### 3.2 Feature selection

In the repeatability analysis, 1,522 radiomic features showed good intra- and inter-observer reliability with ICC values exceeding 0.75 and were retained for further analysis. These features were then subjected to a multi-step feature selection pipeline, in which a combination of ANOVA, Spearman correlation analysis, LASSO and binary logistic regression was applied, resulting in seven predictive radiomic features. The identified features included lbp-2D_glrlm_ShortRunLowGrayLevelEmphasis, log-sigma-1-0-mm-3D_firstorder_RootMeanSquared, wavelet-HHH_glcm_Correlation, log-sigma-1-0-mm-3D_glszm_SizeZoneNonUniformityNormalized, wavelet-HLH_firstorder_Mean, logarithm_gldm_DependenceNonUniformityNormalized, and original_shape_Flatness For the selection of clinical-semantic features, univariable analysis initially identified the following variables as potentially predictive for PTB assessment including satellite nodules, short diameter, age, long diameter, postobstructive pneumonia, vascular invasion, cavitation, lymph nodes, necrosis, sex, calcification and lesion location. After performing multivariate logistic regression analysis, seven clinical-semantic features were found to be statistically significant in predicting PTB. These included lesion location, calcification, vascular invasion, postobstructive pneumonia, satellite nodules, lymph nodes, and sex. Detailed results of the univariable and multivariable analyses are presented in Table 2. To construct the combined model, features selected from both the clinical-semantic and radiomics models were further refined. A final set of five features was identified, including logarithm_gldm_DependenceNonUniformityNormalized, log-sigma-1-0-mm-3D_firstorder_RootMeanSquared, vascular invasion, satellite nodules and lbp-2D_glrlm_ShortRunLowGrayLevelEmphasis. Based on the final logistic regression model, a nomogram for predicting the probability of PTB was constructed, as shown in Supplementary Figure S1.

**Table 2:** Results of univariate and multivariate analysis for the prediction of PTB.

Variable	Univariate analysis		Multivariate analysis	
	OR (95% CI)	*p*-value	OR (95% CI)	*p*-value
Age	<0.001	0.61 (0.48–0.77)		
Sex (female vs. male)	0.029	1.77 (1.06–2.94)	<0.001	0.3 (0.19–0.46)
Location (central vs. peripheral)	0.041	0.29 (0.09–0.95)	0.009	0.15 (0.04–0.62)
Long-axis diameter (mm)	<0.001	0.53 (0.39–0.73)		
Short-axis diameter (mm)	<0.001	0.44 (0.31–0.63)		
Shape (irregular vs. round)	0.190	1.37 (0.85–2.21)		
Calcification	0.029	1.9 (1.07–3.38)	0.008	0.35 (0.16–0.76)
Necrosis	0.025	0.34 (0.13–0.87)		
Cavitation	0.009	2.11 (1.2–3.71)		
Air bronchograms	0.838	1.07 (0.58–1.98)		
Pleural indentation	0.828	1.06 (0.63–1.8)		
Vascular invasion	0.007	0.3 (0.13–0.72)	<0.001	0.1 (0.03–0.32)
Postobstructive pneumonia	<0.001	0.26 (0.12–0.58)	<0.001	0.15 (0.06–0.39)
Satellite nodules	<0.001	172.4 (40.25–738.5)	<0.001	179.79 (37.59–859.84)
Pleural effusion	0.577	1.58 (0.31–7.99)		
Lymph node enlargement	0.011	0.35 (0.15–0.78)	0.037	0.37 (0.14–0.94)

### 3.3 Model performance

**Figure 2:**
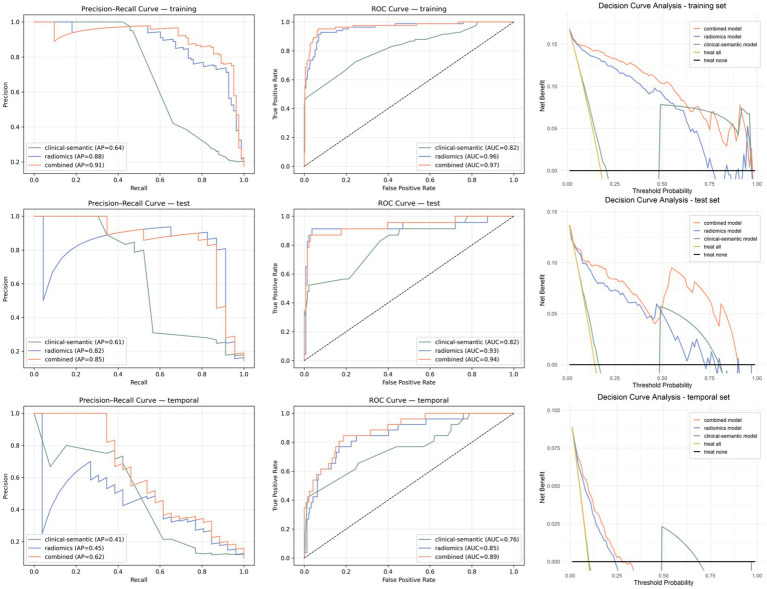
Comparison of model performance for predicting pulmonary tuberculosis across the training, internal validation, and temporal validation sets. Precision-recall curves show model performance in terms of precision and recall, with average precision (AP) used to summarize performance, particularly under conditions of class imbalance. Model discrimination is also measured by the area under the receiver operating characteristic (ROC) curve. Decision curve analysis illustrates the clinical net benefit of each model across a range of threshold probabilities.

The combined model yielded the highest APs across the training, internal validation, and temporal validation sets (0.91, 0.85, and 0.62), significantly outperforming the clinical-semantic model (0.64, 0.61, and 0.41) in all sets (*p* < 0.001, p < 0.001, and *p* = 0.006). The radiomics model also showed good discrimination, with APs of 0.88, 0.82, and 0.45, which were higher than those of the clinical-semantic model across the three sets. Furthermore, the combined model achieved higher F1-scores than the clinical-semantic model (0.84 vs. 0.50, 0.83 vs. 0.40, and 0.46 vs. 0.32 in the training, internal validation, and temporal validation sets, respectively), indicating improved identification of true PTB cases under class imbalance. The F1-scores of the combined and radiomics models were comparable across sets. Besides, NPV, balanced accuracy, and PPV were also calculated for model evaluation. Across all sets, the three models showed consistently high NPVs (0.91–0.99), indicating reliable exclusion of PTB when predictions were negative. Balanced accuracy remained relatively stable for the radiomics and combined models (0.92–0.94 in the training set, 0.91–0.92 in internal validation, and 0.76–0.77 in temporal validation), suggesting that the models maintained good performance across both classes despite class imbalance. As expected in sets with relatively low PTB prevalence, PPV values were comparatively lower. However, the combined model consistently achieved the highest PPV and balanced accuracy across all sets, indicating improved robustness and generalizability compared with the clinical-semantic model alone. Detailed performance metrics are presented in Figure 2 and Table 3.

**Table 3:** Performance metrics of the clinical-semantic, radiomics, and combined models for predicting pulmonary tuberculosis.

Dataset	Model	AP	AUC (95% CI)	Sensitivity	Specificity	PPV	NPV	Balanced-Acc	F1-score	*p*-value
Training	Clinical-semantic	0.64	0.82 (0.76–0.87)	0.72	0.76	0.39	0.93	0.74	0.50	<0.001
	Radiomics	0.88	0.96 (0.93–0.98)	0.93	0.92	0.71	0.98	0.92	0.81	0.084
	Combined	0.91	0.97 (0.94–0.99)	0.95	0.93	0.75	0.99	0.94	0.84	Reference
Internal validation	Clinical-semantic	0.61	0.82 (0.71–0.91)	0.57	0.79	0.31	0.91	0.68	0.40	<0.001
	Radiomics	0.82	0.93 (0.83–1.00)	0.91	0.90	0.62	0.98	0.91	0.74	0.753
	Combined	0.85	0.94 (0.85–0.99)	0.87	0.96	0.80	0.98	0.92	0.83	Reference
Temporal validation	Clinical-semantic	0.41	0.76 (0.65–0.86)	0.65	0.74	0.22	0.95	0.70	0.32	0.006
	Radiomics	0.45	0.85 (0.76–0.93)	0.69	0.85	0.33	0.96	0.77	0.45	0.007
	Combined	0.62	0.89 (0.82–0.94)	0.65	0.87	0.35	0.96	0.76	0.46	Reference

Bootstrap resampling was used to compare the APs of the clinical-semantic and radiomics models against the combined model, which served as the reference.

The clinical utility of the predictive models was evaluated using decision curve analysis. In the training set, the combined model was associated with higher net benefit across a wide range of threshold probabilities (approximately 0.10–0.85) compared with the radiomics model. The radiomics model generally yielded greater net benefit than the clinical-semantic model at lower threshold probabilities (approximately 0.10–0.50). A comparable pattern was observed in the internal validation set, where the combined model maintained relatively higher net benefit across most threshold probabilities. In the temporal validation set, the overall net benefit was relatively lower than that observed in the training and internal validation sets; however, the combined model remained associated with relatively greater clinical benefit within the lower to moderate threshold range. Overall, these findings suggest that the combined model, which integrates radiomic and clinical-semantic information, may provide additional clinical value for decision-making.

To evaluate model calibration, quantitative calibration metrics were calculated, as summarized in Supplementary Table S2. Across all cohorts, the combined model achieved the lowest Brier scores (0.05, 0.06, and 0.12 in the training, internal validation, and temporal validation sets, respectively), compared with the radiomics model (0.07, 0.08, and 0.14) and the clinical-semantic model (0.14, 0.14, and 0.15). The calibration slope for the combined model remains near the ideal value of 1 in the training set (1.03) and shows acceptable calibration in the internal validation set (0.73). Overall, the Brier scores and calibration slopes indicate acceptable calibration of the combined model across the different datasets.

### 3.4 Misclassification of non-tuberculosis pathological types

**Figure 3:**
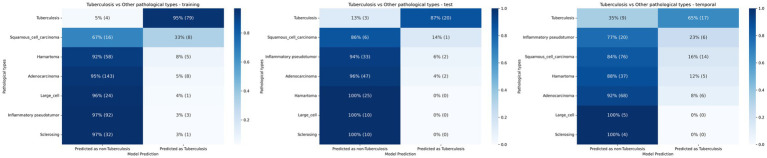
Confusion matrix heatmaps showing the classification results of the combined model for distinguishing pulmonary tuberculosis from non-tuberculosis in the training and validation sets. Each row represents a pathological category, and each cell displays the percentage and count of cases predicted as non-tuberculosis or tuberculosis.

The study also examined non-tuberculosis cases that were misclassified as PTB by the combined model, as illustrated in Figure 3. Squamous cell carcinoma and inflammatory pseudotumor showed relatively higher proportions of misclassification. For squamous cell carcinoma, 33% (8/24) of cases in the training cohort were predicted as PTB, compared with 14% (1/7) in the internal validation cohort and 16% (14/90) in the temporal validation cohort. Inflammatory pseudotumor was misclassified as PTB in 3% (3/95), 6% (2/35), and 23% (6/26) of cases in the training, internal validation, and temporal validation cohorts, respectively. Moderate misclassification was observed for adenocarcinoma and hamartoma. In the case of adenocarcinoma, 5% (8/151) of cases in the training cohort were predicted as PTB, with corresponding proportions of 4% (2/49) in the internal validation cohort and 8% (6/74) in the temporal validation cohort. Hamartoma showed similarly low proportions of misclassification across the cohorts. In contrast, large cell carcinoma and sclerosing lesions showed minimal or no misclassification as PTB, particularly in the validation cohorts.

### 3.5 Model interpretation

**Figure 4:**
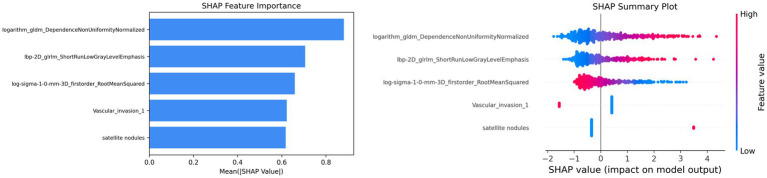
SHAP-based interpretability of the combined model. The left panel shows the SHAP feature importance plot ranking features by their overall contribution to the model. The right panel shows the SHAP summary plot illustrating the direction and magnitude of each feature’s effect on model predictions.

The left panel of Figure 4 presents the SHAP feature importance plot, which ranks variables according to their overall contribution to the predictions of the combined model. The most influential features included the radiomic variables logarithm_gldm_DependenceNonUniformityNormalized, lbp-2D_glrlm_ShortRunLowGrayLevelEmphasis, and log-sigma-1-0-mm-3D_firstorder_RootMeanSquared, as well as the clinical-semantic variables vascular invasion and satellite nodules. The right panel of Figure 4 shows the SHAP summary plot, which illustrates both the magnitude and direction of each feature’s contribution to the model output. Positive SHA*p* values indicate a greater likelihood of classification as PTB, whereas negative values indicate a lower likelihood. Higher values of logarithm_gldm_DependenceNonUniformityNormalized tended to correspond to more positive SHAP values, suggesting that increased texture heterogeneity may be associated with a higher probability of PTB prediction. In contrast, higher values of log-sigma-1-0-mm-3D_firstorder_RootMeanSquared appeared to correspond to more negative SHAP values, indicating a potential association with non-tuberculosis predictions. Besides, the presence of satellite nodules tended to be associated with positive SHAP values, suggesting a higher likelihood of PTB prediction, whereas vascular invasion was more often associated with negative SHAP values, indicating a tendency toward non-tuberculosis classification.

### 3.6 Example cases

**Figure 5:**
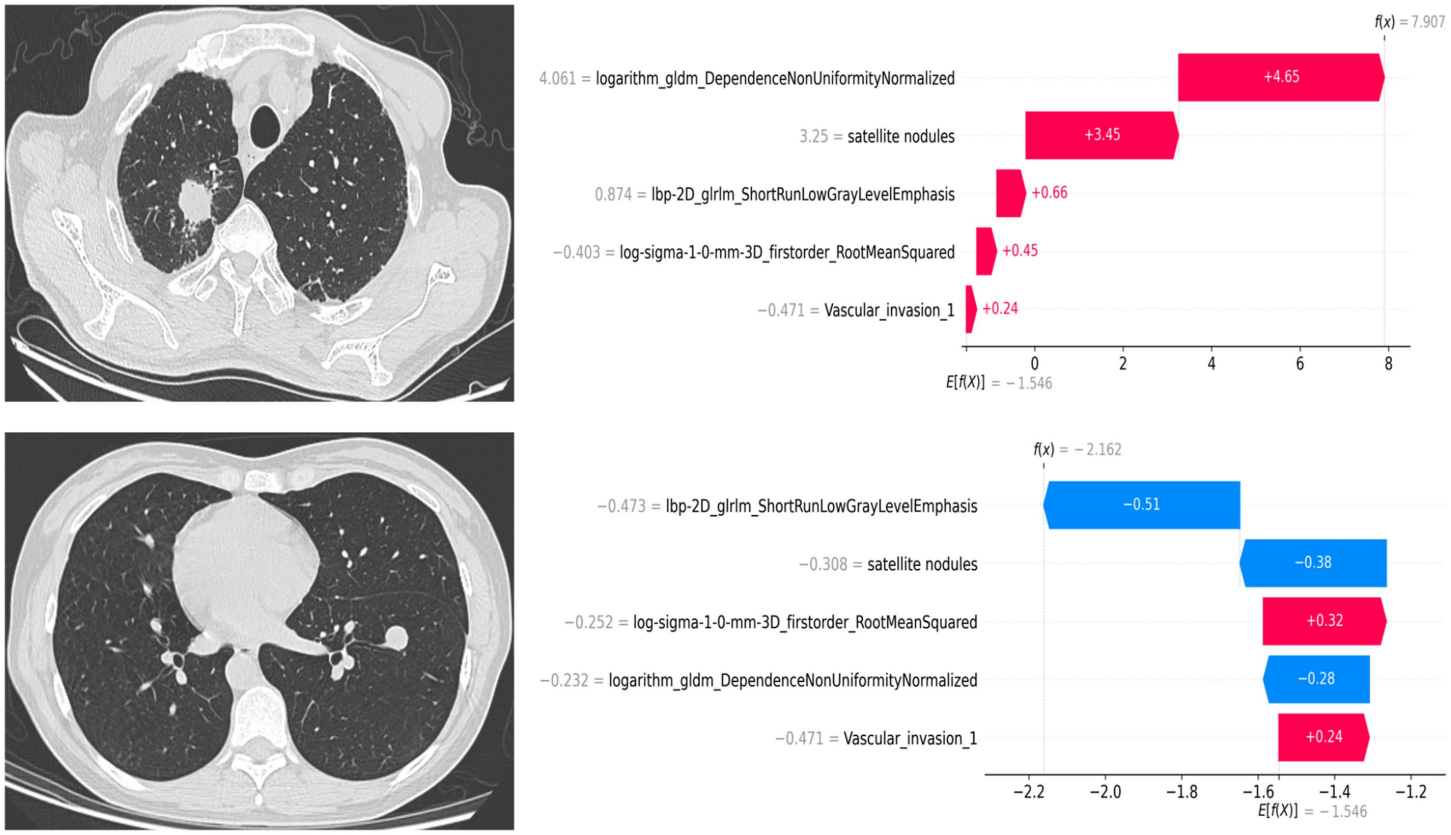
SHAP waterfall plots for case-level interpretation of the combined model. The upper and lower patients were randomly selected as examples of pulmonary tuberculosis and non-tuberculosis, respectively.

To provide insight into case-level interpretability, two patients were randomly selected, and their prediction results were visualized in SHAP waterfall plots (Figure 5). These plots show how individual features contributed positively or negatively to the final predicted outcome.

## 4 Discussion

Accurate prediction of PTB is essential for effective disease control, the formulation of personalized treatment strategies, and informed clinical decision-making. However, the imaging characteristics of PTB often overlap with those of benign and malignant solid lung lesions, making diagnosis challenging. To address this issue, we developed a radiomics model to distinguish PTB from non-tuberculosis cases. The radiomics model achieved good performance, with APs of 0.88, 0.82, and 0.45 in the training, internal validation, and temporal validation sets, respectively. Building upon this, we further investigated whether incorporating clinical-semantic features could enhance model performance. The resulting combined model yielded improved APs of 0.91, 0.85 and 0.62 across the three sets. Additionally, decision curve analysis suggested that both the radiomics model and the combined model may offer clinical benefit across varying threshold probabilities.

Radiomics provides a noninvasive approach for quantitatively characterizing lesion heterogeneity from medical images. In this study, several radiomics features contributed to the discrimination between pulmonary tuberculosis and other solid lung lesions. Among them, higher values of logarithm_gldm_DependenceNonUniformityNormalized were associated with an increased probability of tuberculosis in the model. This feature reflects variability in dependence size within the lesion and may be related to greater internal textural heterogeneity observed in some tuberculosis lesions. In addition, lbp-2D_glrlm_ShortRunLowGrayLevelEmphasis also showed a higher contribution in tuberculosis cases, suggesting a potential association with fine-scale low-intensity textural patterns. Another selected radiomics feature, log-sigma-1-0-mm-3D_firstorder_RootMeanSquared, captures intensity-related information after image filtering and may reflect subtle variations in signal intensity within the lesion. In clinical-semantic features, we found that the presence of satellite nodules was a valuable imaging biomarker for differentiating PTB. This finding was consistent with previous studies based on CT scans (30, 31). Vascular invasion was also included in the final model and may provide additional contextual information regarding lesion morphology and surrounding structures. Together, the integration of radiomics features and clinical-semantic variables may allow the model to capture complementary aspects of lesion appearance, thereby supporting the differentiation between pulmonary tuberculosis and other solid lung lesions.

Researchers have also focused on the differentiation of PTB from other solid lung lesions using radiomics-based approaches. For instance, Zhao et al. developed a radiomics model to distinguish PTB from non-tuberculosis infectious lesions based on 101 CT scans (20). They reported that combining radiomics and semantic features improved model performance, with an AUC of 0.83 compared to 0.71 for the semantic model alone. In our study, the combined model was developed using 474 lesions and achieved a higher AUC of 0.94 in the internal validation set. This performance difference may be attributed to the larger dataset, which allowed for more comprehensive feature extraction and more robust model training. Another study evaluated the utility of integrating radiomics and clinical features to differentiate PTB from adenocarcinoma using 235 PET-CT scans (32). Their combined model also yielded a favorable AUC of 0.91 in the validation set. Although similar performance levels can be reached using PET-based imaging, our study offers a cost-effective alternative based on contrast-enhanced CT, which is more accessible in routine clinical practice.

We further analyzed the patterns of false-positive PTB predictions. In the combined model, a small number of squamous cell carcinomas were predicted as PTB. Clinically, this could initially prompt microbiological testing or additional diagnostic evaluation when tuberculosis is suspected. However, the diagnosis of PTB is rarely established based solely on imaging findings, and persistent diagnostic uncertainty would typically lead to tissue biopsy, thereby reducing the likelihood of substantial delay in cancer diagnosis. In addition, some inflammatory pseudotumors were also predicted as PTB, likely reflecting shared inflammatory imaging characteristics. These cases would usually undergo similar microbiological evaluation and further diagnostic assessment in routine clinical practice. The proposed combined model is intended as a decision-support tool rather than a standalone diagnostic method, and its predictions should be interpreted alongside clinical findings, laboratory results, and radiologist assessment. We recognize several limitations in our study. First, the study only included pathologically confirmed cases, which may introduce spectrum bias, as these patients often represent diagnostically challenging lesions requiring invasive evaluation rather than the full spectrum of pulmonary tuberculosis encountered in routine clinical practice. Future prospective studies incorporating clinically diagnosed TB cases, confirmed by microbiological testing, treatment response, or long-term follow-up, are warranted to further evaluate the model in real-world settings. Second, although CT images from multiple scanners were included, variability in acquisition protocols and scanner settings may influence the reproducibility of radiomic features. In this study, standardized preprocessing and stability-based feature selection were applied to mitigate scanner-related variability. Nevertheless, advanced harmonization approaches, such as ComBat (33), may further reduce scanner-related effects in future multi-center validations. Third, relevant laboratory and clinical variables, such as tuberculosis immunologic tests (e.g., T-cell spot test), inflammatory markers, or prior TB history, were not included in the current analysis. These tests were not consistently available for all patients in this retrospective cohort, which limited their incorporation into the model. Integrating such laboratory indicators and clinical information in future studies may further improve model performance and provide a more comprehensive assessment of tuberculosis risk. Fourth, only contrast-enhanced CT images were included in this study, whereas non-contrast CT is more commonly used in the evaluation of pulmonary tuberculosis in routine clinical practice. Radiomic features derived from contrast-enhanced images may differ from those obtained from non-contrast scans; therefore, the generalizability of the current model to non-contrast CT requires further investigation.

## Data Availability

The raw data supporting the conclusions of this article will be made available by the authors, without undue reservation.

## References

[1] Bagcchi S. WHO'S global tuberculosis report 2022. Lancet Microbe. (2023) 4:e20. doi: 10.1016/S2666-5247(22)00359-7, 36521512

[2] Jeong YJ Lee KS. Pulmonary tuberculosis: up-to-date imaging and management. Am J Roentgenol. (2008) 191:834–44. doi: 10.2214/AJR.07.3896, 18716117

[3] Nachiappan AC Rahbar K Shi X Guy ES Mortani Barbosa EJ Jr Shroff GS . Pulmonary tuberculosis: role of radiology in diagnosis and management. Radiographics. (2017) 37:52–72. doi: 10.1148/rg.2017160032 28076011

[4] Rageade F Picot N Blanc-Michaud A Chatellier S Mirande C Fortin E . Performance of solid and liquid culture media for the detection of *Mycobacterium tuberculosis* in clinical materials: meta-analysis of recent studies. Eur J Clin Microbiol Infect Dis. (2014) 33:867–70. doi: 10.1007/s10096-014-2105-z, 24760249

[5] Yeow K-M Su I-H Pan K-T Tsay P-K Lui K-W Cheung Y-C . Risk factors of pneumothorax and bleeding: multivariate analysis of 660 CT-guided coaxial cutting needle lung biopsies. Chest. (2004) 126:748–54. doi: 10.1378/chest.126.3.748 15364752

[6] Weir-McCall JR Joyce S Clegg A MacKay JW Baxter G Dendl L-M . Dynamic contrast–enhanced computed tomography for the diagnosis of solitary pulmonary nodules: a systematic review and meta-analysis. Eur Radiol. (2020) 30:3310–23. doi: 10.1007/s00330-020-06661-8, 32060716

[7] Weir-McCall JR Debruyn E Harris S Qureshi NR Rintoul RC Gleeson FV . Diagnostic accuracy of a convolutional neural network assessment of solitary pulmonary nodules compared with PET with CT imaging and dynamic contrast-enhanced CT imaging using unenhanced and contrast-enhanced CT imaging. Chest. (2023) 163:444–54. doi: 10.1016/j.chest.2022.08.2227, 36087795 PMC9899635

[8] Jiménez-Serrano S Páez-Carpio A Doménech-Ximenos B Cornellas L Sánchez M Revzin MV . Conventional and contrast-enhanced US of the lung: from performance to diagnosis. Radiographics. (2024) 44:e230171. doi: 10.1148/rg.230171, 38935548

[9] Gillies RJ Kinahan PE Hricak H. Radiomics: images are more than pictures, they are data. Radiology. (2016) 278:563–77. doi: 10.1148/radiol.2015151169, 26579733 PMC4734157

[10] Zheng S Cui X Ye Z. Integrating artificial intelligence into radiological cancer imaging: from diagnosis and treatment response to prognosis. Cancer Biol Med. (2025) 22:6–13. doi: 10.20892/j.issn.2095-3941.2024.0422, 39907115 PMC11795265

[11] Zheng S Liu J Xie J Zhang W Bian K Liang J . Differentiating high-grade patterns and predominant subtypes for IASLC grading in invasive pulmonary adenocarcinoma using radiomics and clinical-semantic features. Cancer Imaging. (2025) 25:42. doi: 10.1186/s40644-025-00864-2, 40155960 PMC11951669

[12] Uhlig A Uhlig J Leha A Biggemann L Bachanek S Stöckle M . Radiomics and machine learning for renal tumor subtype assessment using multiphase computed tomography in a multicenter setting. Eur Radiol. (2024) 34:6254–63. doi: 10.1007/s00330-024-10731-6, 38634876 PMC11399155

[13] Xu Y Lu L L-n E Lian W Yang H Schwartz LH . Application of radiomics in predicting the malignancy of pulmonary nodules in different sizes. Am J Roentgenol. (2019) 213:1213–20. doi: 10.2214/AJR.19.21490, 31557054

[14] Chetan MR Gleeson FV. Radiomics in predicting treatment response in non-small-cell lung cancer: current status, challenges and future perspectives. Eur Radiol. (2021) 31:1049–58. doi: 10.1007/s00330-020-07141-9, 32809167 PMC7813733

[15] Linning E Lu L Li L Yang H Schwartz LH Zhao B. Radiomics for classification of lung cancer histological subtypes based on nonenhanced computed tomography. Acad Radiol. (2019) 26:1245–52. doi: 10.1016/j.acra.2018.10.013 30502076

[16] Fan S Xie J Zheng S Wang J Zhang B Zhang Z . Non-invasive CT based multiregional radiomics for predicting pathologic complete response to preoperative neoadjuvant chemoimmunotherapy in non-small cell lung cancer. Eur J Radiol. (2025) 189:112171. doi: 10.1016/j.ejrad.2025.112171, 40398002

[17] Le VH Kha QH Minh TNT Nguyen VH Le VL Le NQK. Development and validation of CT-based radiomics signature for overall survival prediction in multi-organ Cancer. J Digit Imaging. (2023) 36:911–22. doi: 10.1007/s10278-023-00778-0, 36717518 PMC10287593

[18] Le VH Minh TNT Kha QH Le NQK. Deep learning radiomics for survival prediction in non-small-cell lung cancer patients from CT images. J Med Syst. (2025) 49:22. doi: 10.1007/s10916-025-02156-5 39930275

[19] Yang L Jiang Z Tong J Li N Dong Q Wang K. Development and validation of a preoperative CT-based radiomics nomogram to differentiate tuberculosis granulomas from lung adenocarcinomas: an external validation study. BMC Cancer. (2024) 24:670. doi: 10.1186/s12885-024-12422-3, 38824514 PMC11144314

[20] Zhao W Xiong Z Tian D Wang K Zhao M Lu X . The adding value of contrast-enhanced CT radiomics: differentiating tuberculosis from non-tuberculous infectious lesions presenting as solid pulmonary nodules or masses. Front Public Health. (2022) 10:1018527. doi: 10.3389/fpubh.2022.1018527, 36267999 PMC9577178

[21] Tsao M-S Nicholson AG Maleszewski JJ Marx A Travis WD. Reprint of “Introduction to 2021 WHO Classification of thoracic Tumors”. J Thorac Oncol. (2022) 17:337–40. doi: 10.1016/j.jtho.2022.01.008 35216726

[22] Schober P Vetter TR. Chi-square tests in medical research. Anesth Analg. (2019) 129:1193. doi: 10.1213/ane.0000000000004410 31613806

[23] Applegate KE Tello R Ying J. Hypothesis testing III: counts and medians. Radiology. (2003) 228:603–8. doi: 10.1148/radiol.2283021330, 12881587

[24] DeLong ER DeLong DM Clarke-Pearson DL. Comparing the areas under two or more correlated receiver operating characteristic curves: a nonparametric approach. Biometrics. (1988) 44:837–45. doi: 10.2307/2531595, 3203132

[25] Kramer AA Zimmerman JE. Assessing the calibration of mortality benchmarks in critical care: the Hosmer–Lemeshow test revisited. Crit Care Med. (2007) 35:2052–6. doi: 10.1097/01.ccm.0000275267.64078.b0, 17568333

[26] Lundberg SM Lee S-I (2017). “A unified approach to interpreting model predictions.” In: Proceedings of the 31st International Conference on Neural Information Processing Systems. Long Beach: NeurIPS

[27] Kocak B Baessler B Bakas S Cuocolo R Fedorov A Maier-Hein L . CheckList for EvaluAtion of Radiomics research (CLEAR): a step-by-step reporting guideline for authors and reviewers endorsed by ESR and EuSoMII. Insights Imaging. (2023) 14:75. doi: 10.1186/s13244-023-01415-8 37142815 PMC10160267

[28] Kocak B Akinci D'Antonoli T Mercaldo N Alberich-Bayarri A Baessler B Ambrosini I . METhodological RadiomICs score (METRICS): a quality scoring tool for radiomics research endorsed by EuSoMII. Insights Imaging. (2024) 15:8. doi: 10.1186/s13244-023-01572-w 38228979 PMC10792137

[29] Lambin P Woodruff HC Mali SA Zhong X Kuang S Lavrova E . Radiomics quality score 2.0: towards radiomics readiness levels and clinical translation for personalized medicine. Nat Rev Clin Oncol. (2025) 22:831–46. doi: 10.1038/s41571-025-01067-1, 40903523

[30] Wetscherek MT Sadler TJ Lee JY Karia S Babar JL. Active pulmonary tuberculosis: something old, something new, something borrowed, something blue. Insights Imaging. (2022) 13:3. doi: 10.1186/s13244-021-01138-8, 35001143 PMC8743064

[31] Heo J-N Choi YW Jeon SC Park CK. Pulmonary tuberculosis: another disease showing clusters of small nodules. Am J Roentgenol. (2005) 184:639–42. doi: 10.2214/ajr.184.2.01840639, 15671390

[32] Hu Y Zhao X Zhang J Han J Dai M. Value of 18 F-FDG PET/CT radiomic features to distinguish solitary lung adenocarcinoma from tuberculosis. Eur J Nucl Med Mol Imaging. (2021) 48:231–40. doi: 10.1007/s00259-020-04924-6, 32588088

[33] Orlhac F Eertink JJ Cottereau AS Zijlstra JM Thieblemont C Meignan M . A guide to ComBat harmonization of imaging biomarkers in multicenter studies. J Nucl Med. (2022) 63:172–9. doi: 10.2967/jnumed.121.262464, 34531263 PMC8805779

