# Supplementary material for: Differentiation of pulmonary tuberculosis from non-tuberculous solid lung lesions using radiomics and clinical-semantic features on contrast-enhanced CT
Source: Front Med (Lausanne). 2026 Mar 31;13:1754750. doi: 10.3389/fmed.2026.1754750 (PMC13076353; doi:10.3389/fmed.2026.1754750)
Supplement: Supplementary file 1 [file Table_1.DOCX]

Supplementary Material

# Supplementary Data

Supplementary Table S1 Performance comparison between models.

| **Dataset** | **Model** | **AUC** | **Sensitivity** | **Specificity** | **PPV** | **NPV** | **Balanced-Acc** | **F1-score** |
| --- | --- | --- | --- | --- | --- | --- | --- | --- |
| Train | Logistic Regression | 0.97 | 0.95 | 0.92 | 0.73 | 0.99 | 0.94 | 0.82 |
| Train | Decision Tree | 1.00 | 1.00 | 1.00 | 1.00 | 1.00 | 1.00 | 1.00 |
| Train | SVM | 0.98 | 0.93 | 0.96 | 0.85 | 0.98 | 0.95 | 0.89 |
| Internal Test | Logistic Regression | 0.94 | 0.87 | 0.92 | 0.65 | 0.98 | 0.89 | 0.74 |
| Internal Test | Decision Tree | 0.85 | 0.74 | 0.96 | 0.74 | 0.96 | 0.85 | 0.74 |
| Internal Test | SVM | 0.94 | 0.74 | 0.97 | 0.81 | 0.96 | 0.86 | 0.77 |
| Temporal Test | Logistic Regression | 0.89 | 0.69 | 0.87 | 0.36 | 0.96 | 0.78 | 0.47 |
| Temporal Test | Decision Tree | 0.75 | 0.58 | 0.93 | 0.46 | 0.95 | 0.75 | 0.51 |
| Temporal Test | SVM | 0.86 | 0.62 | 0.92 | 0.44 | 0.96 | 0.77 | 0.52 |

In this study, we also explored machine learning algorithms including decision tree and support vector machine (SVM) models. The detailed results are presented in Table S1. Although these models achieved comparable overall performance, logistic regression showed relatively higher sensitivity for detecting tuberculosis in the validation sets, which is important for identifying tuberculosis among patients with solid lung lesions. In addition, logistic regression provides greater interpretability and facilitates clinical implementation compared with more complex machine learning models. Therefore, considering both the higher sensitivity and the better interpretability of the model, logistic regression was selected as the final modeling approach.


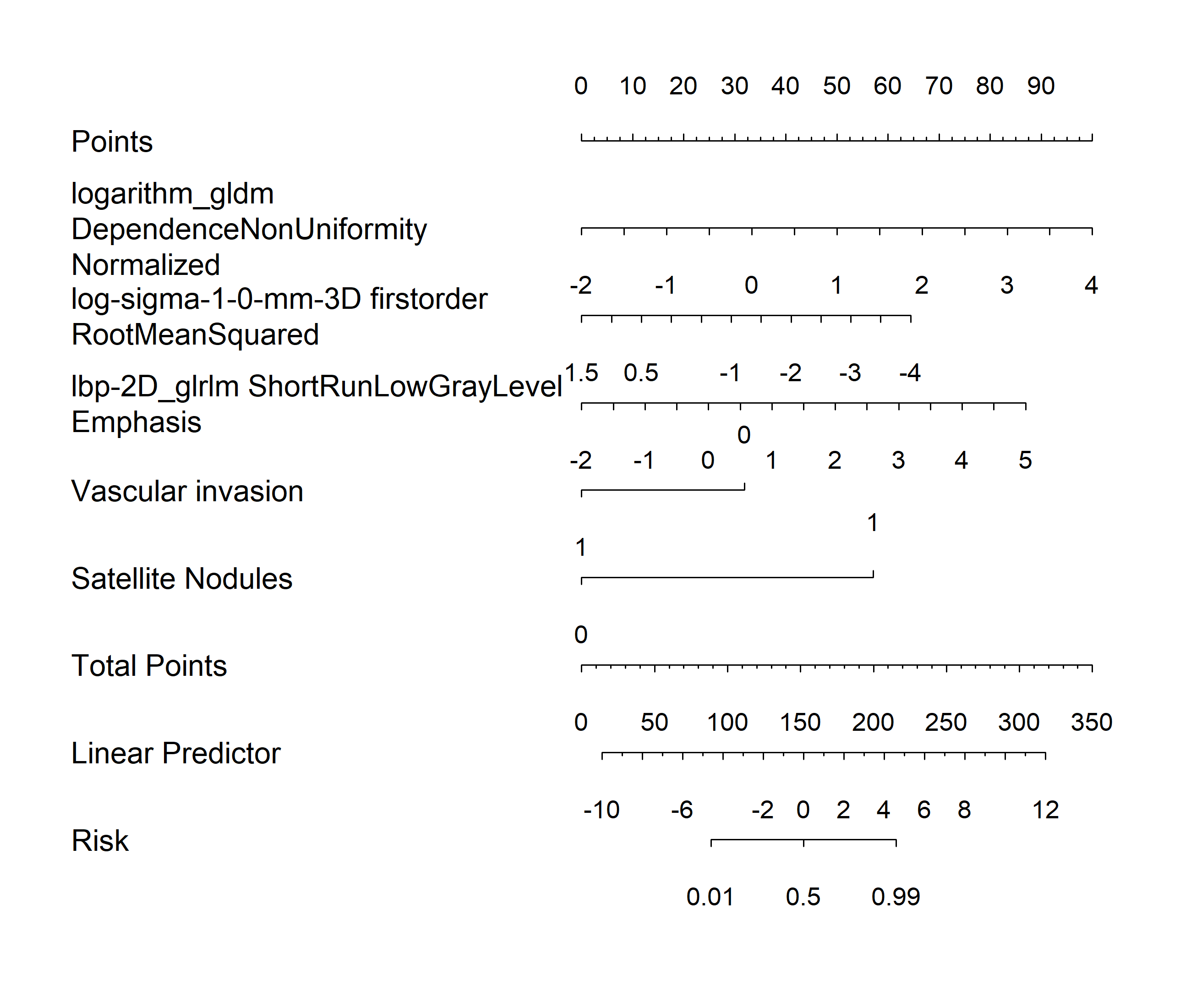


Supplementary Figure S1. Nomogram for predicting the probability of PTB.

The nomogram was constructed based on the final logistic regression model incorporating radiomics features and clinical-sementic variables, including vascular invasion, logarithm_gldm_DependenceNonUniformityNormalized, satellite nodules, log-sigma-1-0-mm-3D_firstorder_RootMeanSquared and lbp-2D_glrlm_ShortRunLowGrayLevelEmphasis. Each predictor corresponds to a point value on the top scale, and the total points correspond to the predicted probability of PTB.

Supplementary Table S2. Calibration performance metrics of the clinical-sementic, radiomics, and combined models across the training, internal validation, and temporal validation cohorts.

| **Dataset** | **Model** | **Brier Score** | **Calibration Slope** | **Calibration Intercept** | **HL Test (p-value)** |
| --- | --- | --- | --- | --- | --- |
| Training | Clinical-semantic | 0.14 | 1.380 | -1.424 | < 0.001 |
|  | Radiomics | 0.07 | 1.030 | -1.545 | < 0.001 |
|  | Combined | 0.05 | 1.033 | -1.491 | < 0.001 |
| Internal Validation | Clinical-semantic | 0.14 | 1.014 | -1.834 | < 0.001 |
|  | Radiomics | 0.08 | 0.906 | -2.028 | < 0.001 |
|  | Combined | 0.06 | 0.730 | -1.779 | < 0.001 |
| Temporal Validation | Clinical-semantic | 0.15 | 1.025 | -2.049 | < 0.001 |
|  | Radiomics | 0.14 | 0.509 | -2.165 | < 0.001 |
|  | Combined | 0.12 | 0.579 | -2.170 | < 0.001 |

Table S2 presents the calibration performance of the three models across all sets. While the Hosmer-Lemeshow test indicated statistical deviation, the test is known to be sensitive to sample size. The combination of low Brier scores and acceptable calibration slopes may indicate the robustness and clinical utility of the integrated diagnostic approach.

**Data inclusion**

The inclusion threshold of 8 mm was based on two primary considerations. According to the Fleischner Society recommendations, pulmonary nodules smaller than 8 mm generally carry a lower risk of malignancy and are typically managed with imaging surveillance rather than immediate diagnostic workup. In routine clinical practice, contrast-enhanced CT is therefore more commonly performed for nodules measuring 8 mm or larger when further diagnostic evaluation is required. As this study included only contrast-enhanced CT examinations obtained during diagnostic assessment and aimed to differentiate pulmonary tuberculosis from malignancy or other non-tuberculous lesions, the analyzed lesions predominantly fell within this clinically actionable size range. In addition, from a radiomics perspective, accurate manual or semi-automated segmentation of nodules smaller than 8 mm can be challenging due to partial volume effects and limited spatial resolution. Excluding these smaller lesions helps reduce feature instability and improves the reproducibility and reliability of radiomic feature extraction.

**Incremental predictive value analysis**

To evaluate whether the addition of radiomic features provided incremental predictive value beyond the clinical-semantic model, we performed Net Reclassification Improvement (NRI), Integrated Discrimination Improvement (IDI), and likelihood ratio testing. The continuous NRI and IDI were calculated by comparing predicted probabilities between the clinical-semantic model and the combined model. Positive values indicate improved classification performance after incorporating radiomic features. In addition, likelihood ratio tests were performed to assess whether the combined model improved model fit compared with the clinical-semantic model. The likelihood ratio statistic was calculated based on the difference in log-likelihood between the two models and compared using the chi-square distribution. The incremental predictive value of radiomic features was evaluated in the internal validation and temporal validation cohorts. In the internal validation cohort, the combined model showed an improvement over the clinical-semantic model, with a continuous NRI of 1.47 and an IDI of 0.35. Likelihood ratio testing also showed the combined model provided a better fit to the data than the clinical-semantic model (χ² = 67.15, p < 0.001). Similarly, in the temporal validation cohort, the combined model achieved a continuous NRI of 1.07 and an IDI of 0.24, indicating improved risk stratification compared with the clinical-semantic model. Likelihood ratio testing again confirmed a better model fit for the combined model (χ² = 38.74, p < 0.001).
